# Pre-Implementation Strategies to Support Adaptation of Thrive: A Care Transitions Model for Economically Disadvantaged Patients with Serious Mental Illness

**DOI:** 10.3390/nursrep14040278

**Published:** 2024-12-02

**Authors:** J. Margo Brooks Carthon, Celsea Tibbitt, Kelvin Eyram Amenyedor, Amanda P. Bettencourt, Erin Babe, Pamela Z. Cacchione, Heather Brom

**Affiliations:** 1 Center for Health Outcomes & Policy Research, Leonard Davis Institute of Health Economics, University of Pennsylvania School of Nursing, 418 Curie Blvd., Philadelphia, PA 19104, USA; ctibbitt@nursing.upenn.edu (C.T.); hmbrom@upenn.edu (H.B.); 2 Yale School of Medicine, Yale University, 333 Cedar St., New Haven, CT 06510, USA; kelvin.amenyedor@yale.edu; 3 Center for Health Outcomes & Policy Research, Penn Implementation Science Center, Leonard Davis Institute of Health Economics, University of Pennsylvania School of Nursing, 418 Curie Blvd., Philadelphia, PA 19104, USA; paamanda@nursing.upenn.edu; 4 Center for Health Outcomes & Policy Research, University of Pennsylvania School of Nursing, 418 Curie Blvd., Philadelphia, PA 19104, USA; erinbabe@upenn.edu; 5 Penn Presbyterian Medical Center, Leonard Davis Institute of Health Economics, University of Pennsylvania School of Nursing, Philadelphia, PA 19104, USA; pamelaca@nursing.upenn.edu

**Keywords:** serious mental illness, Medicaid, transitional support, pre-implementation research, health services research

## Abstract

Background: Economically disadvantaged patients diagnosed with serious mental illness (SMI) experience post-hospitalizations disparities due to fragmented care transitions. Purpose: To describe the pre-implementation strategies used to adapt and implement a nurse-led transitional care intervention (Thrive) to meet the needs of economically disadvantaged patients diagnosed with an SMI. Methods: Two pre-implementation strategies, Evidence Based Quality Improvement (EBQI) meetings and Formative Evaluation (FE) research, were used to adapt intervention components. FE data included semi-structured interviews analyzed using Rapid Qualitative Analysis. Findings: Adaptations were made to core components of Thrive and strategies to support implementation were identified. Conclusions: Participatory strategies help to adapt interventions that are person-centered and tailored to the organizational context. Trial: NCT06203509.

## 1. Introduction

Over 14 million Medicaid-insured adults live with a mental illness, such as major depressive disorder, bipolar disorder, and schizophrenia [1,2]. Medicaid is the largest payer of mental healthcare services, providing coverage for 21% of adults living with a mental illness, and 26% of adults living with a serious mental illness (SMI) [2]. When hospitalized with co-occurring mental and physical health conditions, Medicaid-insured individuals with SMI experience higher rates of readmissions and emergency department (ED) utilization compared with non-Medicaid insured individuals with SMI [3,4,5,6,7,8,9]. These disparities may be related in part to socioeconomic inequities, such as unstable housing, concerns over copays, or barriers in accessing medications [5,7,10,11,12]. Deficits in transitional care support, including limited access to community resources may also contribute to higher rates of readmission and ED utilization [5].

To address the post-discharge needs of people insured by Medicaid our team leveraged principles of human-centered design leading to the development of a clinical innovation called Thrive [13]. Thrive provides intensive wrap-around services including connections to medical and community-based social services to people insured through Medicaid who are transitioning from hospital to home. Now in its fifth year, Thrive has been recognized by the American Academy of Nursing as an Edge Runner for nurse-led innovations. The Thrive model involves an academic clinical partnership between Penn School of Nursing, Penn Medicine Homecare and Hospice services and three large urban hospitals.

A core feature of Thrive is our focus on addressing health-related social needs such as challenges with transportation and food insecurity as a central facet of transitional care support. Services provided through Thrive were designed with the understanding that many disparities experienced by people insured through Medicaid are driven by social and economic factors and the distribution of healthcare resources in such a way that disadvantages some groups over others [14]. Thrive optimizes the post discharge management process by intensifying resources and interdisciplinary collaborations across settings for patients at risk for experiencing health disparities. The published results of Thrive have demonstrated sizable improvements in post-discharge utilization outcomes for Thrive enrollees including higher rates of primary care connections and fewer readmissions and ED visits compared with patients receiving usual care [15].

A notable finding of our early work was the recognition that at least a third of Medicaid-insured participants receiving Thrive services, were also diagnosed with a co-occurring serious mental illness (SMI) [16]. While Thrive services are designed to support non-psychiatric admissions, we frequently witnessed the effects of unstable mental health needs following hospitalization, including a lack of connection to therapy and medication services. Patients with SMI experience a higher burden of medical comorbidities, and frequently follow intricate drug schedules prescribed by multiple healthcare professionals to treat their mental and physical comorbidities. These factors are often complicated by communication difficulties, cognitive impairment, and a lack of social support rendering them more at risk for readmission and ED utilization. Ref. [17] To better understand the experiences of Thrive participants with co-occurring SMI, we undertook a mixed methods pilot study to examine the extent to which Thrive services met the needs of Medicaid-insured patients with co-morbid SMI diagnoses. Findings from the quantitative arm of the pilot study found no significant differences in 30-day ED and readmission rates between Thrive participants with (*n* = 62) or without SMI (*n* = 190). Despite the literature supporting a much higher 30-day (15.9% vs. 11.7%) and 90-day (16.1% vs. 13.2%) readmission rates for those with SMI compared with those without SMI [18,19]. Interviews, however, revealed that while Thrive participants diagnosed with SMI (*n* = 5) expressed satisfaction with services, they reported that their mental health needs were never fully assessed. One participant revealed “*it would have been helpful*” if someone had asked about their mental health needs, but they did not [16].

Like many transitional care programs, Thrive was not developed to meet the specific needs of people diagnosed with SMI. The current literature suggests that many transitional care programs designed for people with SMI focus primarily on support following psychiatric hospitalizations [20]. Components of these programs frequently include transitions of care coach, medication management, and connections to psychiatric providers, though lack attention to health-related social needs such as transportation, food resources, and stable housing. This raises concerns that the transitional care needs of people diagnosed with SMI and hospitalized for non-psychiatric conditions are largely going unmet. For people insured with Medicaid, who experience the additional burdens of economic concerns, the period after hospitalization can be perilous.

Using evidence from our prior findings, we designed a study entitled. “SMI-Thrives: An equity-focused intervention to Improve care transitions for Medicaid insured individuals with co-occurring serious mental health” (hereafter, SMI-Thrives). Using a Hybrid Type I implementation and effectiveness design, and a stepped wedge approach, SMI-Thrives extends and adapts the current Thrive model to address the transitional care needs of patients who are Medicaid-insured, diagnosed with a co-morbid SMI, and hospitalized for a non-psychiatric illness. This article has two aims: First, to describe the pre-implementation evaluation to identify needed components for the adapted Thrive model. Second, we sought to systematically identify barriers and facilitators to the implementation of SMI-Thrive and link them to specific strategies to influence initial adoption.

The SMI-Thrives study is a Type 1 Hybrid Effectiveness-Implementation Stepped Wedge Cluster Randomized Controlled Trial that compares intensive transitional care support to usual care [21]. In the current Thrive model, Medicaid-insured individuals are (1) identified while hospitalized by nurse case managers and referred to home care services using an electronic medical record (EMR) flag. Following discharge, Thrive participants go on to receive (2) home care services where nurses perform medication reconciliation, intensive teaching and chronic disease management. Next, Thrive participants (3) receive continued clinical oversight by our interdisciplinary team including the discharging hospital-based physician or Advanced Practice Provider (APP) who facilitate care continuity if there is an absence of available primary care support. Finally, the nurse led Thrive Care Management Team (4) facilitates weekly interdisciplinary case management meetings for a full month where connections are made to community, primary, and specialty services [22]. The goal of this paper is to describe the pre-implementation processes used by our team to adapt the Thrive care model to meet the transitional care needs of people with co-occurring serious mental health diagnoses hospitalized for a non-psychiatric admission. For our purposes our adaptation followed a thoughtful and deliberate alteration of the design or delivery of Thrive with an aim of improving its fit or effectiveness in a given context [23,24].

We began our pre-implementation process by engaging staff, mental health experts, and community advisors using a participatory process which included a series of “meet and greets” with institutional partners. Next, we launched an Evidence-Based Quality Improvement (EBQI) workgroup to prioritize additional components to the current Thrive care model. To evaluate the context required for the adaptation of the intervention, we used a Formative Evaluation (FE) approach through interviews of clinicians and leadership who would have direct engagement with referrals to Thrive. The University of Pennsylvania Institutional Review Board approved this study.

**Figure 1:**
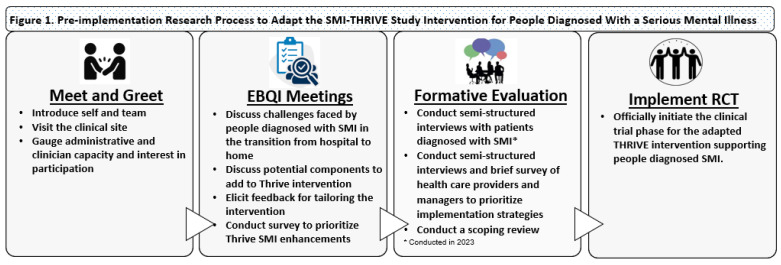
Author’s work. Legend: EBQI—Evidence Based Quality Improvement; RCT—Randomized Controlled Trial, SMI—Serious Mental Illness.

Figure 1 outlines the pre-implementation research process. In the following, we provide an overview of the EBQI and FE approaches. 

## 2. Methods

### 2.1. Evidence-Based Quality Improvement Workgroup

We conducted pre-implementation research using Formative Evaluation (FE) and Evidence-Based Quality Improvement (EBQI) methods to identify additional SMI-sensitive adaptations for the Thrive model and implementation into the trial protocol. The combined strengths of these approaches ensured that the perceptions of diverse stakeholders, including clinicians, administrators, and those diagnosed with SMI, were considered during the adaptation and implementation phases.

### 2.2. EBQI Recruitment and Procedures

The EBQI approach was used to ensure that the added Thrive adaptations were acceptable to stakeholders and consistent with the evidence [25]. For our purposes, the EBQI workgroup members offered perspectives on the challenges faced by patients diagnosed with SMI following hospitalization, and offered suggestions on essential components to consider as adaptations to the Thrive model. Given our goal, we sought workgroup participants such as clinicians with expertise in the care of people diagnosed with SMI, managers from the study site, community representatives, and caregivers of patients with SMI [26]. To ensure we were including perspectives of people with SMI and to protect their privacy, we consulted with two external people diagnosed with SMI who provided feedback throughout the Workgroup process but who preferred not to be identified by their diagnoses. Workgroup members were recruited using both criterion and snowballing techniques which resulted in wide representation.

Our EBQI workgroup first received endorsement from the Hospital Executive team, which helped engender support during workgroup recruitment. The Hospital Executive team was composed of the Nurse Senior Executive and a Clinical Quality Improvement Officer who provided the team with the names of clinicians and managers who could add valuable contributions to the workgroup discussion. In addition, the study team sought the participation of clinicians and content experts (e.g., individuals with experience caring for people diagnosed with SMI) as well as individuals or caregivers of individuals with SMI. The study PI sent personal emails requesting participation in three one-hour work group meetings in early November 2023, with one follow up reminder sent a week after the first. A total of 20 invitations were emailed, with a total of 10 responses. An additional 3 members of the research team were included on the EBQI workgroup bringing the total members to 13. Non-research affiliated workgroup members were provided with a USD 100 honorarium for each one-hour meeting attended.

We hosted three one-hour EBQI workgroup meetings. During meetings, workgroup members offered perspectives on the challenges faced by patients diagnosed with SMI following hospitalization, described current mental health screening practices for non-psychiatric admissions, and prioritized essential components that should be considered as adaptations to the Thrive model. One critical aspect of our work was a review of practices currently operating within the hospital to assess and address the needs of patients diagnosed with co-occurring SMI. Examining existing clinical practices and support systems within the hospital and home care setting helped to set the stage for potential adaptations to the Thrive care model. We aimed to identify gaps and areas for improvement. During each meeting the facilitator stressed the importance of engaging a wide range of perspectives while emphasizing flexible aspects that should be considered. During our final meeting, the workgroup discussed the strengths and merits of 13 possible adaptations to Thrive. Using a consensus approach, participants were asked to rank order their preferences of proposed adaptations. The research team facilitated the consensus process, summarized the results, and then adopted those components that achieved majority support of the 10 non-research workgroup participants.

### 2.3. Formative Evaluation Research

Formative evaluation (FE) is defined as a “rigorous assessment process designed to identify potential and actual influences on the progress and effectiveness of implementation efforts” [26]. For our purposes, FE was used to assess whether Thrive was believed to address the significant needs for patients diagnosed with SMI. In addition, we sought to understand the community and organizational contexts, elicit potential barriers and facilitators, and prioritize implementation strategies to ensure a smooth launch of Thrive at a new site. Our FE processes were informed by the Health Equity Implementation Framework (HEIF), an adaptation of the Integrated Promoting Action on Research in Implementation in Health Services [i-PHARIS] framework [27]. The FE included semi-structured interviews of clinicians and administrators who would be directly involved in the referrals and clinical care management of patients enrolled to Thrive. The HEIF framework proposes determinants that are believed to predict successful and equitable implementation of an intervention. The three health equity domains of HEIF are cultural factors, clinical encounter factors, and societal contexts. Engaging in this participatory process allowed us to increase buy-in from stakeholders and overcome barriers prior to the launch of the study.

### 2.4. Semi-Structured Interview Recruitment

We used a criterion sampling framework to select interview participants. Criterion sampling involves choosing a setting, group, and/or individuals because they represent one or more criteria [28]. For our purposes, we were most interested in gaining the perspectives of individuals who would have direct interface in referring to Thrive, providing clinical oversight or managerial support. Hence, participants deemed eligible for semi-structured FE interviews included case managers, social workers, and healthcare providers affiliated with the study hospital or home care setting. Eligible participants included those who had been employed with the healthcare system for at least 3 months.

### 2.5. Semi-Structured Qualitative Interviews

The formative evaluation (FE) qualitative data were derived from interviews conducted virtually via Teams. Interviews were collected between February and March 2024, after the piloting of the interview guide by two study team members and authors (MBC, HB). Interview questions, informed by the Health Equity Implementation Framework, asked participants to reflect on perceived appropriateness of Thrive, challenges faced by patients following hospitalization, anticipated barriers to intervention acceptance, and organizational and workflow challenges, if any (Table 1). Interviewers obtained verbal consent to record the interviews and memos were taken throughout the duration of the encounter. Twelve eligible participants were contacted by email for interviews and a total of eight agreed to be interviewed, which is sufficient to elicit rich and in-depth perspectives from study participants [16,29].

*Procedures.* All eligible participants were contacted by email. The recruitment email included a link where potential participants could access information about the study and a consent document and select dates for interviews. Once the interview time was selected, the participant received confirmation by email and a unique Teams link. Recruitment emails were sent in three waves at the beginning of the study and in two-week increments as a reminder. A reminder email was sent 24 h prior to each interview. Each interview lasted approximately 40 min and was followed by a brief 5 min survey that asked for demographics, and 36 questions related to preferred implementation strategies and feasibility of the Thrive intervention. Detailed memos were taken throughout the interviews by the research program manager (KA). Twelve of these items included Proctor’s implementation outcomes that assess the acceptability (how fair or reasonable Thrive is deemed), appropriateness (to what extent Thrive seems suitable), and feasibility (the practicality and ease of delivering Thrive). Each construct is four-items assessed on a five-point Likert scale asking participants to rate how much they agree or disagree with each item. The items are averaged with higher scores indicating greater acceptability, appropriateness, or feasibility [30]. The survey also asked participants to rank the usefulness of 12 implementation strategies drawn from the ERIC taxonomy. Participants were asked to select either “most useful” or “least useful” for each potential implementation strategy. Finally, readiness for change was assessed using the 12-item Organizational Readiness for Implementing Change instrument. Participants rated their level of agreement or disagreement with statements about their organization on a five-point Likert scale. The items are averaged with higher scores indicating more agreement that the organization is ready for change [30].

*Reflexivity Statement.* Our interview team purposely included individuals with expertise in equity and implementation science. One interviewer is Black, and the other is White. Both interviewers have background clinical experience working across hospital and community settings and with patients with chronic illnesses including SMI. Half of the authorship team identify as persons of color and all have substantial research experience in the fields of equity, mental health, implementation science, and nursing. Weekly meetings held throughout the interview process included discussion on how our social and clinical identities or closely held assumptions may influence our interpretation of participant’s responses.

### 2.6. Semi-Structured Interview Data Analysis

*Structured template and matrix display.* A multistep process was used to summarize interview recordings using rapid qualitative analysis (RQA) procedures [25,31]. The RQA process is a well-established process ideal for use during the pre-implementation process when a deductive approach is in use and when evaluating the feasibility or appropriateness of an intervention. Using Hamilton’s and Finley’s, 2019, method, we began by identifying interview responses corresponding to each interview question [31,32,33]. Our interview guide was developed based on the domains of HEIF. Interview questions explored participant’s views on the appropriateness of Thrive, aspects of the clinical encounter that might influence acceptance of Thrive, workflow facilitators and barriers, as well as community and organizational context.

Qualitative team members read the transcripts and noted particularly rich responses that were detailed and meaningful. We then developed a structured template to standardize the way to capture the interview content. The domains of HEIF were listed on the template for each study team member (HB and MBC) to summarize interview key ideas. To ensure inner rater reliability and applicability to the interview, two study team members used the template to separately code the same transcripts. The summaries of the two team members were used to confirm the relevancy of the template by comparing and modifying the template as needed. The two team members then divided the remaining interviews and summarized them using the template and HEIF domains. Responses that entailed representative quotes were timestamped for future exploration [34].

The researchers mapped each template onto a visual matrix after summarizing each transcript. The visual matrix consists of a single column per interview transcript, with each HEIF domain represented by a row. Once the findings were entered onto the visual matrix, each row was interpreted, and key ideas were synthesized to assess convergence and divergence across participants.

Rigor was established by adhering to the four dimensions for qualitative research outlined by Guba and Lincoln [35] (1981) which include the following: credibility (e.g., training interviewer with relevant expertise), confirmability (e.g., leveraging methodological triangulation using a brief follow up survey), dependability (e.g., maintaining a detailed audit trail), and transferability (developing an operations definition to achieve saturation).

## 3. Results

### 3.1. Evidence-Based Quality Improvement (EBQI) Workgroup Demographics

The EBQI workgroup included thirteen participants, six of whom were female. Four participants were Black, two identified as White, and two identified as other. The educational level of the workgroup members ranged from a baccalaureate degree to doctoral degree. The workgroup consisted of one SMI caregiver, two SMI healthcare professionals, one home care nurse manager, two case managers, two physicians, one advanced practice provider, one quality improvement officer, and three researchers.

### 3.2. FE Interview Participant Demographics

There were eight interview participants, each with an average of 2 years of experience in their current positions. All participants were female, four participants were Black, three participants were White, and one participant identified as “other”. The highest level of education attained was a doctoral degree. Other characteristics of the formative evaluation (FE) participants are detailed in Table 2.

### 3.3. Evidence-Based Quality Improvement (EBQI) Workgroup Discussion Results

The EBQI workgroup held a total of three meetings. In the first meeting, the current Thrive model was introduced, and participants shared their current transitional care challenges for people diagnosed with SMI. Chief concerns raised during the discussion included the following: a lack of care integration/coordination, limited access to outpatient mental health services, need for medication management and reconciliation of psychiatric meds, mistrust and stigma, training healthcare professionals, need for formal mental health screening. The focus of the second meeting included proposed adaptations to the existing Thrive model. Examples of proposed adaptations included the following: addition of mental health screening during discharge process and integrated behavioral health assessments during Thrive case management meetings. The third and final EBQI workgroup meeting used a consensus process to decide on the proposed elements of the final adapted Thrive model. Table 3 displays the workgroup priorities and level of agreement with proposed elements of the Thrive model.

### 3.4. Evidence-Based Quality Improvement (EBQI) Workgroup Members Endorsed the Following Elements to the Adapted Thrive Model

The team discussed a wide range of potential adaptations to the Thrive intervention to support the needs of patients diagnosed with SMI. Of the seven potential adaptations, the three that received at least 2/3 endorsements by EBQI members included ensuring the comprehensive integration of a mental health assessment during the Thrive weekly interdisciplinary team meetings. This involved creating a standardized inclusive checklist pertaining to all mental health diagnoses, documenting the status (active vs. remission) of mental health diagnoses, and determining current behavioral treatments or levels of engagement with a mental health provider. Participants also endorsed the need to integrate mental health screening into discharge planning during afternoon team huddles. Finally, recognizing that some participants would be hesitant to receive Thrive or home care services, it was proposed that a member of the Thrive team conduct bedside visits to share further information about Thrive.

### 3.5. Formative Evaluation (FE) Results

Table 4 provides an overview of the FE results analyzed using rapid qualitative analysis and based on the domains of the HEIF. These domains include perceptions of the Thrive innovation, the needs of eligible intervention recipients, community resources, and organizational, healthcare system, and provider factors.

Interview participants provided perspectives about the challenges faced by potential Thrive recipients, including unaddressed complex social needs such as high levels of co-occurring SMI and substance-use disorder. Post-discharge barriers were frequently assessed (e.g., lack of housing, transportation, financial concerns, food insecurity), yet connections to community resources, primary care providers, and mental health services were often lacking. Of note, interview participants voiced that eligible patients may be resistant to Thrive services due to fear or mistrust. One case manager noted,

*“… you can offer people referrals to community resources, community health workers, substance abuse and they’re like ‘no’”.*

*“[They] don’t want people in their homes. Fear that people are going to judge them”.*

When speaking to the context of the health system and community connections, participants reported fractured coordination to community resources and a disconnect between the front-line staff and senior leadership. Participants noted the unequal distribution of resources between hospitals within the same system. While simultaneously noting there are overlapping programs and a lack of coordination among those that have similar aims.

*“I think the elephant in the room really is how the communication between home health and the hospitals. How can we make that a little bit more fluid”*

(Case Manager)

Participants also described a disconnect between the context of the community in which patients diagnosed with SMI currently live and the resources they need post-discharge. Another case manager noted,

*“I still don’t think as a health system we understand how people in this community really live and what they don’t have”.*

FE interview participants also revealed their perceptions of provider-specific factors contributing to transitional care challenges for patients with SMI. Some participants disclosed limited understanding of the social barriers faced by many patients which may influence the way they are able to connect with patients faced with SMI or economic challenges. One case manager noted,

*“I think as healthcare workers we have this perception that patients come to us for a service and they should follow our rules and do what we say but that’s not life and most of our providers and clinicians and nurses, we can’t relate to how they live outside of the hospital so it’s no way you can say abide by their rules when we don’t know what their life is like”.*

When considering any barriers or facilitators to implementing Thrive, participants conveyed overwhelming support. They noted that Thrive appeared to be a low-burden addition to their inpatient workflow. Participants noted that Thrive was well-aligned with organizational priorities already in place including reducing readmissions and ED use. Participants were enthusiastic about improving continuity of care through the intervention.

*“I really think that having something like this [Thrive], even if you can’t save all, you’ll be able to save a lot more than what we’re able to do now”.*

(Social Worker)

Interview participants were asked to provide their preferences for up to 13 implementation strategies. Proposed strategies and level of endorsement are detailed in Table 5. Of the thirteen potential strategies, six were unanimously endorsed including identifying champions, audit and feedback, quarterly updates with leadership, educational meetings and training, train-the-trainer strategies, and the inclusion of a reminder flag in the EHR. Strategies that received unanimous support from interview participants were discussed during EBQI workgroup meetings and integrated into the launch of the Thrive intervention.

## 4. Discussion

Our use of an Evidence-Based Quality Improvement (EBQI) workgroup and Formative Evaluation (FE) approaches allowed us to engage a wide range of stakeholders to inform the adaptation and implementation of Thrive in a new clinical setting. Through this process of participatory engagement, we successfully adapted the Thrive model to suit the needs of Medicaid-insured patients diagnosed with a co-occurring SMI and discharged from a non-psychiatric hospitalization.

Our final adaptation of the Thrive model was informed by the QUERI Roadmap for Implementation and Quality Improvement (2020) which outlines the following steps for adaptation, (1) assessing the fit to ensure that the adaptation of Thrive met the needs of our local setting, (2) assessing the importance of the adaptation to end users, (3) including stakeholders in what and how to adapt Thrive, and (4) making final adaptations prior to launching the trial [21]. The workgroup discussion and interview results conveyed the shared sentiments of perceived value and the need for the services offered through Thrive. Our team sought input from end-users and those affected by SMI to support each step of the adaptation and implementation strategy selection process resulting in the revised Thrive care model.

### 4.1. THRIVE Final Adapted Model

**Figure 2:**
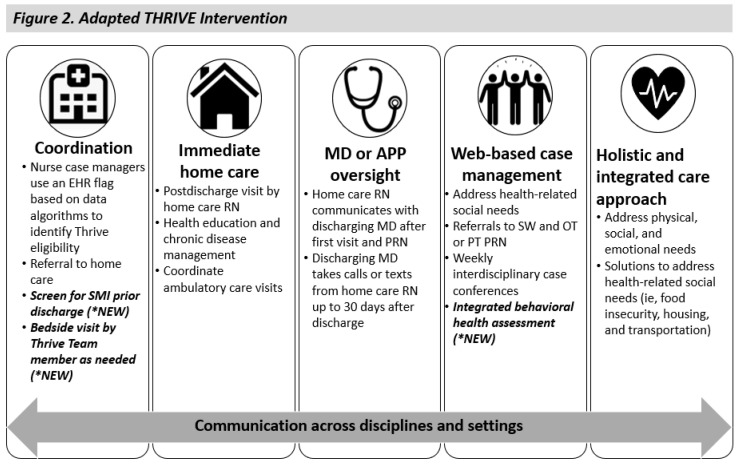
Adapted from Brooks Carthon et al. “Transitional care innovation for Medicaid-insured individuals: early findings” [15].

The adapted Thrive model has several new key processes and core components. The first process includes the conduct of a screening for serious mental illness prior to hospital discharge (see Figure 2). The mental health screen is completed by a Nurse Case Manager or Social Worker and incorporated into the readiness assessment that all patients receive as a part of discharge planning. Next, because mistrust was identified as a potential threat to acceptance as well as a lack of time by social workers and case managers to fully explain the benefits of Thrive, a second process adaptation was added to the Thrive model, namely “bed-side visits’, conducted by a Thrive team member. During bed-side visits, the Thrive virtual care coordinator or program manager visits eligible patients during hospital admission to provide an in-depth overview of the Thrive program and to answer any questions. This approach was believed to offer the benefits of more time for engagement and question answering and to relay experiences of prior Thrive participants. In addition to bedside visits, case managers and social workers reported other strategies to engage and increase trust among patients with SMI. They incorporate normalizing questions about mental health as routine, like other physical health-related questions. They also encouraged patients to exercise their agency in centering their personal goals in the discharge planning process. Finally, a key core component to the Thrive model includes the inclusion of a comprehensive behavioral health assessment during weekly virtual case conferences. This additional assessment by the Thrive care team includes a careful review of all mental health diagnoses and psychiatric medications, including the last date filled and the prescriber. A note with this information is sent to the social worker and the need for further mental health intervention is reviewed. The level of connection to a current mental health provider is assessed and, if needed, referrals for mental health support are provided.

Finally, consistent with recent calls for integrating implementation science and readiness for change into pre-trial planning [36,37,38], our FE and EBQI workgroup helped guide the use of implementation strategies to implement and support the successful launch of this study. Of note, selected implementation strategies included the following: identifying and preparing champions who were defined as dedicated clinicians embedded in the health system that could support, market, and drive the Thrive intervention. These individuals were viewed as stakeholders who could help overcome resistance in the organization. For our purposes, a Social Work Manager was selected as a Thrive champion who would bring Thrive up during daily team meetings and help to answer any questions raised by colleagues. A second implementation strategy included providing audit and feedback sessions to clinicians monthly. This feedback was viewed as a useful way to monitor, evaluate, and course correct as needed. Quarterly meetings with health system leadership were also endorsed as a way to ensure that the project remains aligned with strategic initiatives. The addition of interactive and educational meetings with staff involved in implementing Thrive—using train the trainer sessions—to facilitate Thrive peer training were offered to ensure training was accessible and credible. Finally, the inclusion of an EHR Thrive reminder flag was endorsed by all participants as a tool to embed a reminder into usual workflows.

### 4.2. Limitations

There are several limitations to our use of EBQI and FE approaches. First, we were limited to individuals connected to a single hospital and homecare setting situated in a large northeast setting. Hence, the perspectives of necessary resources, challenges, and necessary Thrive adaptations and implementation strategies may vary by clinical setting and geographical region. However, future research should be multi-site to increase generalizability and could consider adapting the Thrive model for patients experiencing a psychiatric hospitalization. Finally, we conducted a relatively small number of FE interviews. Interviewing a larger number, though not necessary for qualitative research, could have nonetheless yielded deeper insights on implementation processes and perceived barriers.

## 5. Conclusions

Our use of a rigorous pre-implementation process helped adapt the nurse-led Thrive transitional care model for people who are economically disadvantaged and diagnosed with a serious mental illness. These participatory approaches helped engage individuals with diverse viewpoints and resulted in intervention and implementation strategies, that are person-centered and tailored to the organizational context.

## Figures and Tables

**Table 1:** Health Equity Implementation Framework-informed interview guide.

Domain	Example Interview Questions
**Clinical Encounter**	What types of difficulties do patients with significant social needs and/or SMI encounter after hospitalization?
**Cultural Factors**	Considering the challenges in accepting services that carry stigma, like behavioral health resources, what tactics or communication techniques do you find most effective in motivating patients to agree to post-discharge support upon discharge?
**Societal Context**	We understand that your hospital has a unique organizational structure with the health system. Are there any unique community collaborations that would support (or hinder) your ability to implement the clinical intervention?

**Table 2:** Interview Participant Demographics (*n* = 8).

Variable	
**Age, mean (SD)**	38.9 (11.3)
**Years at current job setting, mean (SD)**	2.0 (1.4)
**Female, *n* (%)**	8 (100%)
**Race *n* (%)**	
Black or African American	4 (50%)
White	3 (37.5%)
Other	1 (12.5%)
**Ethnicity, *n* (%)**	
Hispanic	0
**Highest level of education, *n* (%)**	
Baccalaureate degree	1 (12.5%)
Master’s degree	5 (62.5%)
Doctoral degree	1 (12.5%)
MD	1 (12.5%)
**Primary job, *n* (%)**	
Case manager	2 (25.0%)
Social worker	2 (25.0%)
Physician	1 (12.5%)
RN clinical practice lead	1 (12.5%)
Hospital nurse manager	1 (12.5%)
Home care nurse	1 (12.5%)

**Table 3:** Workgroup Priorities for Seven Identified SMI-focused Enhancements to the Thrive Intervention.

Intervention Enhancements, %	Very Important	Important	Somewhat Important	Not Important
Comprehensive integration of mental health into weekly Thrive virtual management calls (e.g., create a formal checklist that reviews mental health diagnosis and current treatment).	71.4%	28.6%	-	-
Integration of mental health in discharge planning (e.g., discussing Thrive during afternoon team huddles)	71.4%	28.6%	-	-
In-person discussions for patients who initially refuse services	71.4%	14.3%	14.3%	-
Presentation of Thrive information to Thrive-eligible individuals with a focus on physical, SDOH, and mental health: elevator pitch by case managers and social workers	57.1%	42.9%	-	-
Trauma-informed care as part of Thrive training	42.9%	42.9%	14.2%	-
Care package that focuses on self-care and mental health concept	28.6%	71.4%	-	-
Thrive training of staff nurses	14.3%	85.7%	-	-

**Table 4:** Results of rapid qualitative analysis based on the domains of the Health Equity Implementation Framework.

HEIF Domain	Summary	Exemplar Quote
**Characteristics of the Innovation:** The extent to which the innovation is simple (ease of use), intuitive, and considered to be beneficial or useful (effectiveness). How the proposed integration of the Thrive referral or follow up by APPs or Hospitalists would increase (time) or change workflow	Overwhelming support from all participants to institute Thrive Low-burden addition to the inpatient workflow Aligned with organizational priorities including reduced readmissions and ED utilization Enthusiastic about improving continuity of care through the intervention Intervention viewed as necessary since many patients have co-occurring serious mental illness (SMI)	“I really think that having something like this [Thrive], even if you can’t save all, you’ll be able to save a lot more than what we’re able to do now” (Participant 3)
**Clinical Encounter**: Clinical workflows leading to interactions between patient and provider or between recipients. Includes consideration of unique characteristics and preferences affecting engagement with populations of interest and techniques used by providers to gain trust/improve communication	Very important to build patient–provider trust so that patients will be open to accepting a home care/Thrive referral Shorter lengths of stay can be a barrier to fully understanding all the patient’s health-related social needs once discharged Clinicians found it helpful to use communication techniques that met the patient where they were—e.g., good eye contact, normalizing their health or mental health condition	“’you know what’s best for you’, so encouraging and reminding patients that they have the autonomy and decision” (Participant 2) “there’s a care team that’s responsible for you but there’s a person on this team and that person is you, like you’re a part of it and so you have to do that part. You need people who aren’t afraid to have those conversations” (Participant 3)
**Patient Factors**: Specific to a patient or member of the healthcare team and can refer to believes, acceptance, training and knowledge communication, or engagement preferences. Also includes attitudes towards relevant stakeholders of institutions	Many complex social needs that remain unaddressed Lack connections to community resources (e.g., primary care providers, mental health service providers) High levels of co-occurring serious mental illness and substance use disorder May be resistant to services/providers they do not already know Many barriers post discharge (e.g., lack of housing, transportation, caregiver, financial concerns) High burden placed	“we’ve assisted with like keeping their electricity on but they don’t necessarily do their part to kind of make sure that they stay up to date with things;” (Participant 3) “We know you can offer people referrals to community resources, community health workers, substance abuse and they’re like ‘no’” (Participant 2) “Don’t want people in their homes. Fear that people are going to judge them” (Participant 2) “Need support for caregivers—they are burdened with family member’s complexity” (Participant 8)
**Provider Factors**: Specific to a member of the healthcare team and can refer to believes, acceptance, training and knowledge communication or engagement preferences. Also includes atttudes towards relevant stakeholders of institutions	Providers unable to attend to all of the needs patients have Needing to meet patients where they are in understanding their life circumstances Risk of burnout and compassion fatigue when caring for patients who can’t follow discharge recommendations related to a lack of community services/supports Frustration that despite efforts to connect patients to resources that patients are still returning repeatedly to the hospital Limited expertise to meet the needs of people diagnosed with co-occurring SMI and SUD	“Sometimes all we can do is give a list of resources” (Participant 1) “I think as healthcare workers we have this perception that patients come to use for a service and they should follow our rules and do what we say but that’s not life and most of our providers and clinicians and nurses, we can’t relate to how they live outside of the hospital so it’s no way you can say abide by their rules when we don’t know what their life is like” (Participant 3) “mix of like some patients have tremendous support and other don’t, so, it can feel, it can certainly contribute to burn out. It can certainly contribute to ambivalence about how effective we are … when we suspect or worry that many of the recommendations won’t be able to be done for the various barriers that the patients are facing” (Participant 5)
**Context**: Inner/outer (community/health system/organization factors) Outer: Formal policies, previous experiences specific to the hospital Inner: What factors related to the hospital may have influenced the success of prior interventions Organizational level: community hospital, merger with a larger health system, safety net Community and Local: metro/urban area in the northeast, availability of behavioral health resources Healthcare system—neighborhood clinics, relationship with HUP—Spruce	Fractured community resources Long-wait times for mental health connections once discharged to the community Co-located FQCH seen as a strength Great external resources (PMHC) Disconnection between the front line and senior leadership related to the necessary resources to meet patient needs Unequal distribution of resources between hospitals in the same system Overlapping programs and lack of coordination among programs that have similar aims	“I still don’t think as a health system we understand how people in this community really live and what they don’t have” (Participant 3) “I think the elephant in the room really is how the communication between home health and the hospitals. How can me make that a little bit more fluid” [Participant 8]
**Societal factors**: Structural and economic factors that may significantly affect healthcare disparities and implementation drivers, including federal or state, or local policies	New CMS policies requiring SDOH screen has increased the focus on health-related social needs and making connections	

**Table 5:** Usefulness Ratings of Implementation Strategies.

Proposed Implementation Strategies	Hypothesized Mechanism of Action	Most Useful	Least Useful
Identify and Prepare Champions	People who will dedicate themselves to supporting, marketing, and driving the intervention, overcoming resistance in an organization. Those who expressed curiosity at first and immediately saw the benefit to patients of Thrive.	100%	0
Audit and Feeback to Clinicians	Giving periodic feedback at regular intervals helps monitoring, evaluation, and course correction as needed	100%	0
Quarterly Meetings with Leadership	Align project with strategic initiatives	100%	0
Conduct Educational Meetings	Information dissemination and knowledge on process for patient referral	100%	0
Train the Trainer Strategies	Designating clinicians to train peers makes education accessible and credible	100%	0
EHR Flag in Electronic Medical Record	The flag reminds clinicians to refer to Thrive	100%	0
Develop Academic Partnerships	Bidirectional relationship—research, coordination, data analytic and clinical skills, local knowledge, passion, influence	85.7%	14.3%
Provide Professional Incentives	Making clinician more engaged, feeling fulfilled in the work they do	85.7%	14.3%
Email Reminders Sent after Initial Orientation Training	The email helps clinicians recall training information and act on it	85.7%	14.3%
Use Mass Media: Use of Educational Flyers and Buttons, etc.	Items spread the word about the innovation among clinicians	85.7%	14.3%
Place Info. About Thrive into Hospital-wide Townhall Hosted by CEO or Board Meetings	Influential opinion leader promotes program and shares success	85.7%	14.3%

## Data Availability

We will preserve and share de-identified data products with the Inter-university Consortium for Political and Social Research (ICPSR) data repository. We will follow the comprehensive de-identification standards set forth in the ICPSR “Guide to Social Science and Data Preparation and Archiving, 6th Ed” for both quantitative and qualitative data, including the removal of any direct identifiers, date-shifting of indirect identifiers (e.g., dates of service), creating pseudonyms, and collapsing/combining variables that might potentially be identifiable (e.g., clinician roles) in the context of the entire qualitative transcript corpus.

## Public Involvement Statement

No public involvement in any aspect of this research.

## Guidelines and Standards Statement

This manuscript was drafted against the Standards for Reporting Implementation Studies (StaRI) Statement for reporting implementation science studies (Pinnock, H.; Barwick, M.; Carpenter, C.R.; Eldridge, S.; Grandes, G.; Griffiths, C.J.; Rycroft-Malone, J.; Meissner, P.; Murray, E.; Patel, A.; et al. Standards for Reporting Implementation Studies (StaRI) statement. *BMJ* **2017**, *356*, i6795).
